# Standards of NGS Data Sharing and Analysis in Ataxias: Recommendations by the NGS Working Group of the Ataxia Global Initiative

**DOI:** 10.1007/s12311-023-01537-1

**Published:** 2023-03-04

**Authors:** Danique Beijer, Brent L. Fogel, Sergi Beltran, Matt C. Danzi, Andrea H. Németh, Stephan Züchner, Matthis Synofzik, AGI Ataxia NGS genomics, platforms Working Group, Astrid Adarmes, Saud Alhusaini, Mahmoud Reza Ashrafi, Luis Bataller, Enrico Bertini, Sylvia Boesch, Ronald Buijsen, Emanuel Cassou, Edwin Chan, Joana Damásio, Karina Donis, Ewelina Elert-Dobkowska, Liena Elsayed, Carmen Espinos, Haşmet Hanağasi, Morteza Heidari, Wolfgang Nachbauer, Jorge Oliveira, Puneet Opal, Coro Paisan-Ruiz, Hélène Puccio, Francesco Saccà, Maria Luiza Saraiva-Pereira, Thorsten Schmidt, Rebecca Schüle, Giovanni Stevanin, Carlo Wilke, Grace Yoon, Neta Zach, Ginevra Zanni

**Affiliations:** 1 Dr. John T. Macdonald Foundation Department of Human Genetics and John P. Hussman Institute for Human Genomics, University of Miami Miller School of Medicine, Miami, FL USA; 2 Division Translational Genomics of Neurodegenerative Diseases, Hertie-Institute for Clinical Brain Research and Center of Neurology, University of Tübingen, Hoppe-Seyler-Strasse 3, Tübingen, Germany; 3 Departments of Neurology and Human Genetics, David Geffen School of Medicine, University of California Los Angeles, Los Angeles, CA USA; 4 CNAG-CRG, Centre for Genomic Regulation (CRG), The Barcelona Institute of Science and Technology, Baldiri Reixac 4, 08028 Barcelona, Spain; 5 Universitat Pompeu Fabra (UPF), Barcelona, Spain; 6 Departament de Genètica, Microbiologia I Estadística, Facultat, de Biologia, Universitat de Barcelona (UB), 08028 Barcelona, Spain; 7 Nuffield Department of Clinical Neurosciences, University of Oxford, Oxford, UK; 8 Oxford Centre for Genomic Medicine, Oxford University Hospitals NHS Foundation Trust, Oxford, UK; 9 Center for Neurodegenerative Diseases (DZNE), Tübingen, Germany

**Keywords:** Consensus, Genomics, Cerebellar ataxia, High-throughput nucleotide sequencing, Information dissemination

## Abstract

The Ataxia Global Initiative (AGI) is a worldwide multi-stakeholder research platform to systematically enhance trial-readiness in degenerative ataxias. The next-generation sequencing (NGS) working group of the AGI aims to improve methods, platforms, and international standards for ataxia NGS analysis and data sharing, ultimately allowing to increase the number of genetically ataxia patients amenable for natural history and treatment trials. Despite extensive implementation of NGS for ataxia patients in clinical and research settings, the diagnostic gap remains sizeable, as approximately 50% of patients with hereditary ataxia remain genetically undiagnosed. One current shortcoming is the fragmentation of patients and NGS datasets on different analysis platforms and databases around the world. The AGI NGS working group in collaboration with the AGI associated research platforms—CAGC, GENESIS, and RD-Connect GPAP—provides clinicians and scientists access to user-friendly and adaptable interfaces to analyze genome-scale patient data. These platforms also foster collaboration within the ataxia community. These efforts and tools have led to the diagnosis of > 500 ataxia patients and the discovery of > 30 novel ataxia genes. Here, the AGI NGS working group presents their consensus recommendations for NGS data sharing initiatives in the ataxia field, focusing on harmonized NGS variant analysis and standardized clinical and metadata collection, combined with collaborative data and analysis tool sharing across platforms.

Inherited ataxias are among the neurological disorders with the highest genetic and disease burden, as well as a significant genetic heterogeneity. Extensive implementation of next-generation sequencing (NGS) in clinical and research settings has vastly increased the identification of gene loci associated with ataxia, with now > 200 primary ataxia-associated genes identified [1–3]. Despite the increased identification of causal ataxia genes and mutations, currently ~ 50% of patients with hereditary ataxia remain genetically undiagnosed [2, 4–6]. This gap is problematic as identification of the underlying genetic cause provides ataxia patients with an etiological diagnosis of their disease, and thereby facilitates counselling about disease features and prognostic disease trajectories. The identification of the genetic cause in respective patients is nowadays also the entry point to potential targeted genetic therapies for an increasing number of ataxias [3, 7].

As part of the Ataxia Global Initiative (AGI) [8], the AGI working group for “ataxia NGS genomics and platforms” aims to improve the methods, platforms, and international standards for ataxia NGS analysis and data sharing thereby increasing the number of ataxia patients for whom a genetic ataxia diagnosis can be established. Identification of *PNPLA6* as a molecular cause for Gordon Holmes and Boucher-Neuhäuser ataxia syndromes (Fig. 1) exemplifies the success of global collaborative data sharing and analyses of ataxia NGS datasets from Europe, and North and South America on a capable NGS platform (GENESIS) [9]. This involved the following key elements: (i) NGS sequencing of unsolved ataxia patients at disseminated sites in different continents (Europe, North America, South America); (ii) collaborative sharing of these NGS datasets from different sites in one joint collaborative NGS platform (GENESIS); (iii) collaborative NGS analysis of these shared NGS datasets within the web-based NGS platform, allowing both decentralized analysis and work on the datasets at each individual site as well as joint centralized NGS analysis; and (iv) hereby leveraging two different analysis strategies in parallel: a standardized variant analysis protocol (see below), and a striking phenotypic syndrome shared by all affected subjects as a seed for the analysis (Boucher-Neuhäuser Syndrome, Gordon-Holmes Syndrome (Fig. 1)). Ideally, new genetic ataxia conditions represent druggable targets for which trial-readiness and natural history studies can be performed [8, 10]. To set the stage for a large-scale, collaborative international endeavor in the field of ataxia genomics, the AGI NGS working group is utilizing and harmonizing major ataxia NGS databases in the field, facilitating accessibility to NGS datasets across major NGS ataxia partners, and providing NGS analysis toolsets for ataxia NGS analysis, including standardized filter criteria and analysis routines.

**Fig. 1:**
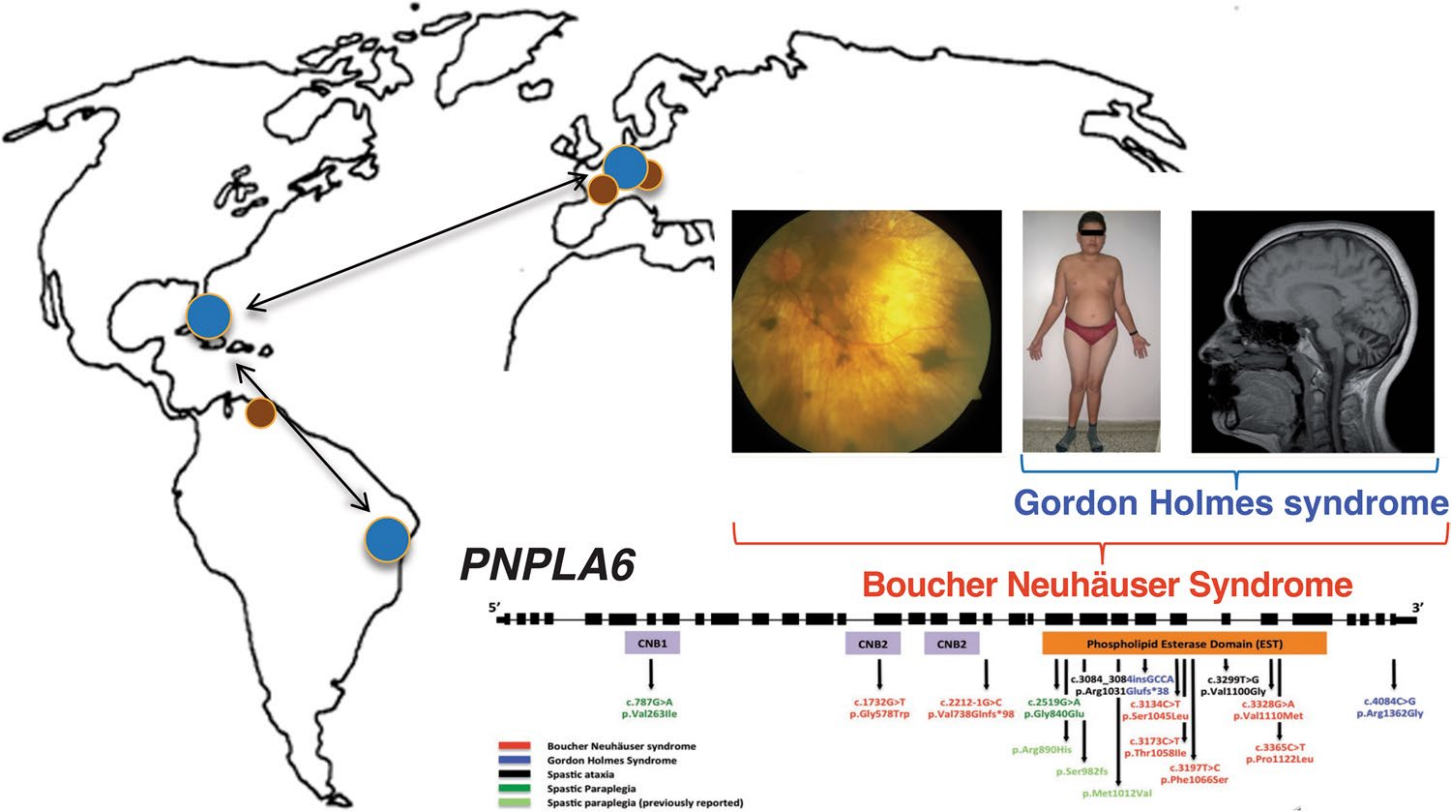
Discovery of *PNPLA6* gene causative for ataxia-disorders through collaborative data sharing efforts. *PNPLA6* was identified as a novel genetic cause for Gordon Holmes and Boucher-Neuhäuser ataxia syndromes through the successful global data sharing and analyses of ataxia NGS datasets from Europe, and North and South America on a capable NGS platform (GENESIS)

In preparation of this effort, the working group has generated, analyzed, and facilitated NGS data sharing of ataxia patients across ataxia NGS platforms and sites around the world, with unprecedented global coverage (> 4700 ataxia datasets, > 75 clinical sites on 6 continents). Here, we describe a series of consensus standards, jointly developed by the AGI NGS working group (including coordinators of three major international collaborative ataxia NGS platforms) for (1) standardized ataxia NGS analysis, (2) standardized clinical (meta-)data collection, and (3) recommendations and resources for international ataxia NGS data sharing initiatives.

## The Current Challenges in Ataxia NGS Genomics

**Fig. 2:**
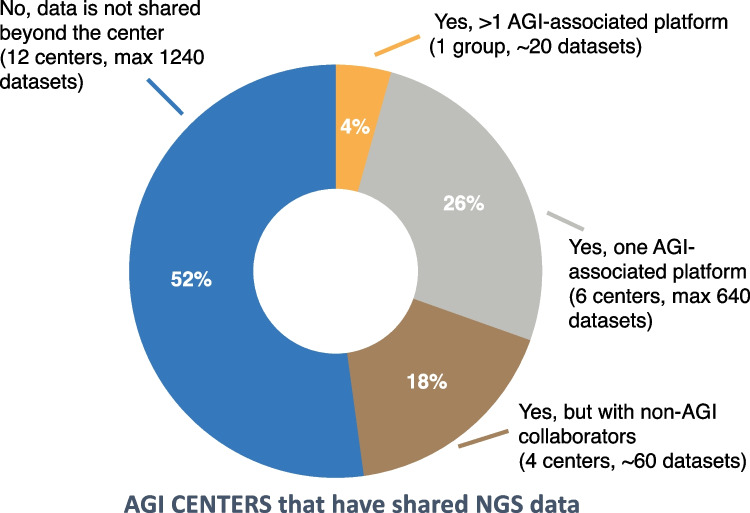
Sharing of NGS data by AGI centers. Results from an October 2021 survey among AGI associated centers showing that from all centers with ataxia NGS data > 50% of centers do not yet share the NGS datasets beyond their own center, which also makes up the majority of datasets

The AGI NGS working group is effectively targeting two of the main roadblocks in the field of ataxia genomics: (1) rare ataxia patients are scattered across specialized centers around the world, with the number of patients seen per center and clinician typically being low and (2) the ataxia NGS datasets in different research groups often remain effectively siloed. This is evidenced by an AGI survey in October 2021 of 32 AGI-affiliated ataxia centers with NGS data around the world, for which the survey aimed to assess where and if NGS data is shared with external groups and/or platforms. The survey results demonstrated the siloing of NGS data effectively with > 70% of research groups not yet sharing their NGS datasets with any of the main ataxia NGS databases and > 50 not sharing the NGS data with any external platforms (Fig. 2).

GENESIS, GPAP, and GAGC—the main AGI-associated ataxia NGS data sharing platforms—represent efforts to counter this international fragmentation of ataxia NGS data, by providing harmonized and accessible platforms for data submission and analysis. This has led to the largest aggregation of ataxia NGS data worldwide at a cumulative total of > 4700 datasets [11, 12]. All three platforms combine standardized machine-readable phenotypic data in combination with genomic data, and have been highly successful in discovering novel ataxia genes and providing genetic diagnoses. Table 1 provides further details on the characteristics of these NGS data sharing platforms.

**Table 1:** Overview of important metrics, capabilities, and characteristics of the AGI-affiliated ataxia NGS platforms

	GENESIS (www.tgp-foundation.org/genesis-log-in)	RD-Connect GPAP (https://platform.rd-connect.eu/)	CAGC (https://uclahs.fyi/cagc)
Platform Go-live date	2011	2015	2020
# of ATX WGS/ total NGS datasets	147 / 2060	287 / 1898	61 / 789
% (#) solved ATX NGS datasets	7.6% (158)	9.8% (186 user reported)	25.4% (201)
# novel ATX genes discovered (notable examples)	> 17 genes (*POLR3A, PNLPA6*)	> 16 genes	*TRPC3*
# ATX publications supported by the platform	40 +	Number not actively tracked	20 +
# of unique partners	100 +	60 groups (specifically on ATX datasets)	20 +
# of total registered users for ATX NGS datasets	130 +	91 users have submitted data	Number not actively tracked
# of users querying the platform at least once since 1/1/2020	105	195 individual users	Over 60% of registered users have active queries
Accepted data formats for upload	Genomics (BAM, FASTQ, CRAM, VCF) Metadata/phenotype (XLS, TXT)	Genomics (BAM, FASTQ, CRAM), Phenomics (JSON, XLS, PhenoPackets), Metadata (User interface or XLS)	Genomics (FASTQ, BAM/CRAM, VCF) Metadata/phenotype (XLS, TXT)
Pipeline includes the following: WES, WGS, panels, SNV, SV, ROH, repeat expansions, other (list what applies)	WES, WGS, panels, microVCFs SNV, SV, CNV, ROH, Indels, repeat expansions, relatedness, ancestry, mitochondrial variants	WES, WGS, SNV, CNV, ROH, Indels	WES, WGS, CNV, ROH, repeat expansions, relatedness, ancestry
Server architecture	Central server architecture on AWS	Central server architecture on owned servers	Central server architecture on AWS and owned servers
Data sharing policy	Opt in; enforced for specific consortia (PREPARE)	Enforced after optional embargo period. Option to share with specific users/projects during embargo period. Sharing of queries and variant tags	Opt in
HIPAA-compliance for clinical NGS datasets?	No. PHI/PII not accepted for upload. Compliant with GDPR	No. It is compliant with EU GDPR	Yes

*ATX*, ataxia; *AWS*, Amazon Web Services; *BAM*, Binary Alignment Map; *CNV*, copy number variation; *CRAM*, Compressed Reference-oriented Alignment Map; *GDPR*, General Data Protection Regulation; *HIPAA*, Health Insurance Portability and Accountability Act; *JSON*, JavaScript Object Notation; *NGS*, next-generation sequencing; *ROH*, runs of homozygosity; *SNV*, single nucleotide variant; *SV*, structural variant; *VCF*, variant call format; *WES*, whole exome sequencing; *WGS*, whole genome sequencing

## Consensus Recommendations for Standardized Ataxia NGS Annotation, Filter Settings, and Genome Analysis

The general recommendation is to transition research and clinical grade molecular genetic analysis to whole genome sequencing (WGS) approaches, when cost effective to do so. The high-fidelity gold standard is currently the Illumina platform of short-read sequencing instruments, although the use of long-read whole genome sequencing at genome scale is increasing within research settings. By relying on different techniques, long-read sequencing—Oxford Nanotech Technology (ONT) or Pacific Biosciences (PacBio)—is more accurate for detection of larger structural variants (SVs) or repeat expansions (as frequent in ataxia, e.g. *RFC1* [13] or GAA-*FGF14* [14]), but still requires further advances to match accuracy and cost for single-nucleotide variants (SNVs). Beyond the detection of SNVs and small insertions and deletions, bioinformatic advances increasingly support calling of short-tandem repeats (STRs), structural variants, and copy-number variations in short-read NGS. This reduces the need for locus-specific molecular tests (e.g. gene panels). The use of advanced techniques—WGS over WES and long-read over short-read—should be prioritized whenever reliably possible, as these types of datasets will have broader utilization and are useful for reanalysis for many years to come supporting novel bioinformatic approaches. However, collection of multigene panels, single STR evaluations, and clinical and full exome data, remain important for pathogenic allele frequency estimations, allelic series collection, positive controls for novel WGS-based analyses (e.g. long-read sequencing, optical genome mapping, bioinformatic algorithms), and simply as research documentation. Although genomic analysis recommendations are focused on research, the use of accepted clinical standards is encouraged. These include the ACMG (American College of Medical Genetics and Genomics) criteria for variant pathogenicity and the ClinGen frameworks for gene and variant classification [15, 16].

### Annotations

Key variant annotations for the analysis of large ataxia datasets should include the following:

- chromosomal position with genome reference build and consensus transcript (for standardized reidentification and comparison)
- variant class/type
- inheritance/variant zygosity
- allele frequencies in in-house and large population databases (e.g. gnomAD v2.1.1.) frequency and allele count
- pathogenicity predictions (e.g. CADD, GERP)
- quality criteria (coverage or read depth, quality score, genotype confidence)

In the future, it would be recommended to fully annotate the standardized ACMG classification criteria for each individual variant.

### Filter Settings

**Fig. 3:**
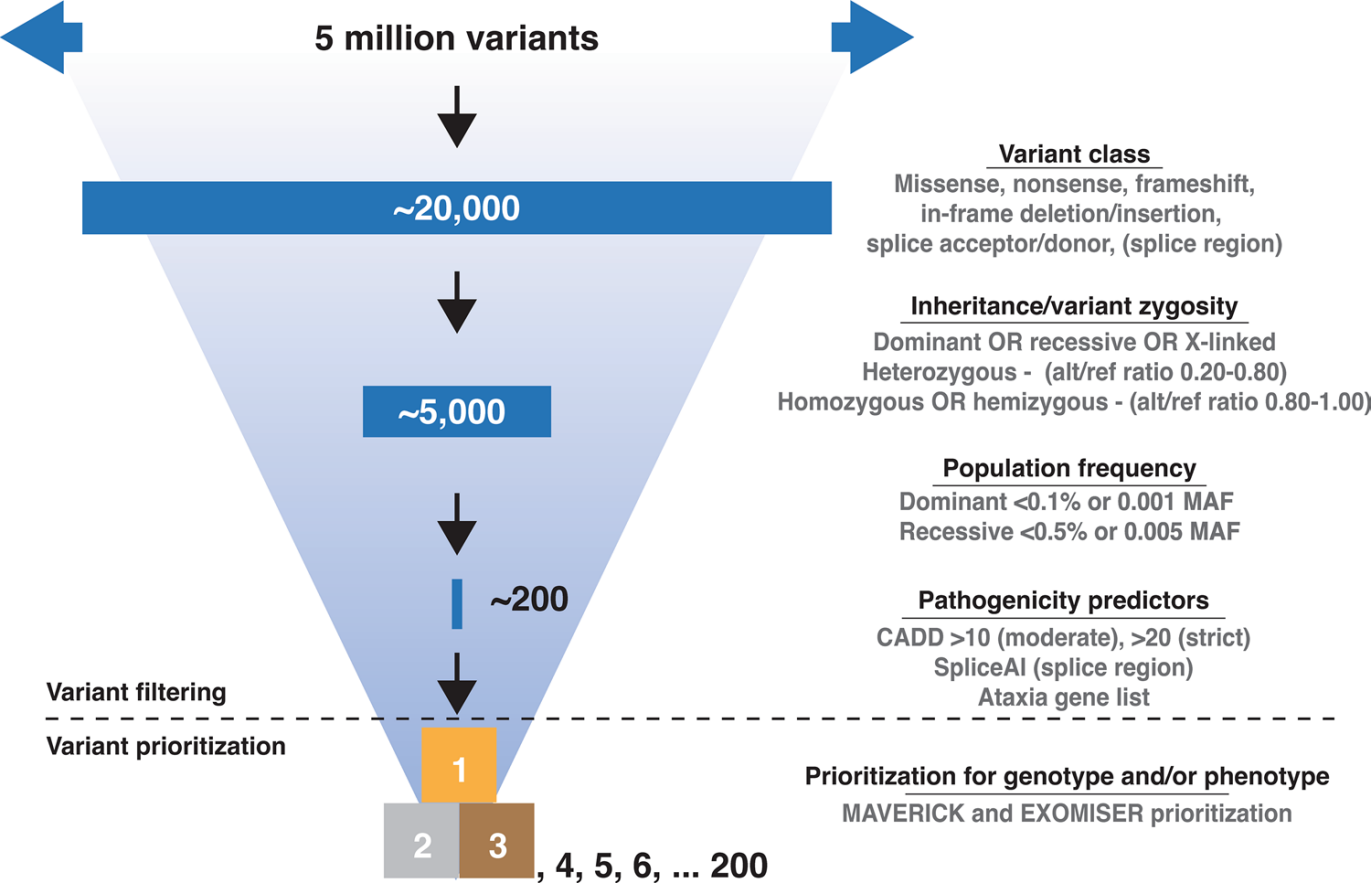
NGS filter and prioritization settings. WGS for a single individual will yield over 5 million variants requiring filtering to identify the causal pathogenic variant. Variant filtering depends on several steps of filtering (1) variant class, (2) inheritance/variant zygosity, (3) population frequency, and (4) pathogenicity predictions (e.g. CADD, GERP), SpliceAI. Lastly, as a final step of variant prioritization, Exomiser and Maverick (AI-tools) can be applied

To allow for minimal standards of ataxia NGS analysis in the field, a common core set of variant filter settings should be used for analyzing ataxia NGS datasets. These are research-use filter recommendations, differing from interpretation guidelines suggested by clinical advisory variant classification frameworks. Pathogenic variants are primarily those that (have the potential to) affect the translated protein (missense, nonsense, frameshift, in-frame deletion/insertion, splice acceptor/donor, and splice region). Causal variants should not be technical artifacts (alt/ref ratios) and should fit with the observed inheritance pattern (zygosity, dominant/recessive/X-linked/mitochondrial). Pathogenic variants should also occur less frequently in the general population than the (rare) disease phenotype (allele frequencies). As, the last step in variant filtering, variant pathogenicity prediction scores can be used (e.g. CADD, GERP, spliceAI). We have summarized our recommendations for autosomal dominant and recessive filter settings in Fig. 3.

Settings might also include a filter for variants in known ataxia genes, allowing users to find “low-hanging fruit” variants early in the NGS analysis process, preventing them from being overlooked. For this, the AGI NGS working group has compiled a current minimum set of 383 ataxia and ataxia-spectrum genes in Table 2. Such an *in silico* panel of known ataxia genes will also help to identify new mutations, thus far not associated with the known mutational mechanism or allelic spectrum of a given gene, e.g. SCA27/*FGF14* locus and the newly discovered intronic repeat expansion [14] or a complex structural variant identified in the ataxia gene *GLS* [17].

**Table 2:** List of ataxia-associated genes, containing core ataxia genes and ataxia-spectrum genes, for use in filtering NGS data

Gene set (*n* = 389) (ataxia plus ataxia-overlap diseases, including all genes from gene set #1)
*ABCB7*, *ABCD1*, *ABHD12*, *ACO2*, *ADCK3*, *AFG3L2*, *AHI1*, *AIMP1*, *ALAS2*, *ALDH18A1*, *ALDH3A2*, *ALG6*, *ALS2*, *AMACR*, *AMPD2*, *AMT*, *ANG*, *ANO10*, *AP4B1*, *AP4E1*, *AP4M1*, *AP4S1*, *AP5Z1*, *APTX*, *ARG1*, *ARL13B*, *ARL3*, *ARL6IP1*, *ARMC9*, *ARSA*, *ARSI*, *ARX*, *ATCAY*, *ATL1*, *ATM*, *ATP13A2*, *ATP1A3*, *ATP2B2*, *ATP2B3*, *ATP2B4*, *ATP7B*, *ATP8A2*, *AUH*, *B4GALNT1*, *B9D1*, *BEAN1*, *BICD2*, *BSCL2*, *BTD*, *C10ORF2*, *C12orf65*, *C19orf12*, *C5orf42*, *CA8*, *CACNA1A*, *CACNA1G*, *CACNB4*, *CAMTA1*, *CAPN1*, *CC2D2A*, *CCDC88C*, *CCT5*, *CEP104*, *CEP120*, *CEP290*, *CEP41*, *CLCN2*, *CLN5*, *CLN6*, *CLN8*, *COQ2*, *COQ6*, *COQ8A*, *COQY*, *CP*, *CPT1C*, *CSF1R*, *CSPP1*, *CSTB*, *CTDP1*, *CWF19L1*, *CYP27A1*, *CYP2U1*, *CYP7B1*, *DAB1*, *DARS*, *DARS2*, *DDB2*, *DDHD1*, *DDHD2*, *DLAT*, *DNAJC19*, *DNAJC3*, *DNAJC5*, *DNM2*, *DNMT1*, *DSTYK*, *EEF2*, *EIF2B1*, *EIF2B2*, *EIF2B3*, *EIF2B4*, *EIF2B5*, *ELOVL4*, *ELOVL5*, *ENTPD1*, *EPM2A*, *EPT1*, *ERCC2*, *ERCC3*, *ERCC4*, *ERCC5*, *ERLIN1*, *ERLIN2*, *ETFA*, *ETFB*, *ETFDH*, *EXOSC3*, *FA2H*, *FAM126A*, *FAM134B*, *FARS2*, *FBXO7*, *FGF14*, *FIG4*, *FLRT1*, *FLVCR1*, *FOLR1*, *FUS*, *FXN*, *GAD1*, *GALC*, *GAN*, *GARS1*, *GBA*, *GBA2*, *GBE1*, *GCDH*, *GCH1*, *GCLC*, *GCSH*, *GFAP*, *GJB1*, *GJC2*, *GLB1*, *GLDC*, *GLRX5*, *GLTP*, *GM2A*, *GNB1*, *GOSR2*, *GPR56*, *GPT2*, *GRID2*, *GRM1*, *HACE1*, *HEPACAM*, *HERC1*, *HEXA*, *HEXB*, *HPRT1*, *HSD17B4*, *HSPD1*, *HTRA1*, *HYLS1*, *IBA57*, *IFIH1*, *IFRD1*, *IGDCC3*, *INPP5E*, *INPP5K*, *IRF2BPL*, *ITM2B*, *ITPR1*, *KCNA1*, *KCNA2*, *KCNC1*, *KCNC3*, *KCND3*, *KCNJ10*, *KIAA0226*, *KIAA0556*, *KIAA0586*, *KIAA0753*, *KIDINS220*, *KIF1A*, *KIF1C*, *KIF5A*, *KIF5C*, *KIF7*, *KLC2*, *KLC4*, *KY*, *L1CAM*, *L2HGDH*, *LYST*, *MAG*, *MAN2B1*, *MARS1*, *MARS2*, *MFN2*, *MFSD8*, *MKS1*, *MLC1*, *MME*, *MRE11*, *MRE11A*, *MSTO1*, *MT-ATP6*, *MTPAP*, *MTRR*, *MTTP*, *NARS2*, *NDUFS7*, *NEU1*, *NHLRC1*, *NIPA1*, *NKX2-1*, *NKX6-2*, *NOL3*, *NPC1*, *NPC2*, *NPHP1*, *NT5C2*, *NUBPL*, *OFD1*, *OPA1*, *OPA3*, *OPTN*, *PANK2*, *PAX6*, *PCNA*, *PCYT2*, *PDE6D*, *PDHX*, *PDSS1*, *PDSS2*, *PDYN*, *PEX1*, *PEX10*, *PEX11B*, *PEX12*, *PEX13*, *PEX14*, *PEX16*, *PEX19*, *PEX2*, *PEX26*, *PEX3*, *PEX5*, *PEX6*, *PEX7*, *PGAP1*, *PHYH*, *PIBF1*, *PIK3R5*, *PLA2G6*, *PLEKHG4*, *PLP1*, *PMM2*, *PMPCA*, *PNKP*, *PNPLA6*, *POLG*, *POLH*, *POLR3A*, *POLR3B*, *PPT1*, *PRDX3*, *PRICKLE1*, *PRKCG*, *PRPS1*, *PRRT2*, *PTRH2*, *RAB3A*, *RAB3GAP2*, *RARS2*, *REEP1*, *REEP2*, *RELN*, *RFC1*, *RNASEH2B*, *RNASET2*, *RNF168*, *RNF170*, *RNF216*, *RNU12*, *RPGRIP1L*, *RRM2B*, *RTN2*, *SACS*, *SCYL1*, *SEC16A*, *SERAC1*, *SETX*, *SIL1*, *SKOR1*, *SLC16A2*, *SLC17A5*, *SLC19A3*, *SLC1A3*, *SLC25A15*, *SLC25A46*, *SLC2A1*, *SLC33A1*, *SLC39A8*, *SLC52A2*, *SLC9A1*, *SLC9A6*, *SMPD1*, *SNAP25*, *SNX14*, *SOD1*, *SOX10*, *SPAST*, *SPG11*, *SPG20*, *SPG21*, *SPG7*, *SPR*, *SPTAN1*, *SPTBN2*, *SQSTM1*, *STUB1*, *SUN1*, *SUN2*, *SYNE1*, *SYT14*, *TARDBP*, *TBCC*, *TBCE*, *TCTN1*, *TCTN2*, *TCTN3*, *TDP1*, *TDP2*, *TECPR2*, *TFG*, *TGM6*, *TH*, *TMEM138*, *TMEM216*, *TMEM231*, *TMEM237*, *TMEM240*, *TMEM67*, *TPP1*, *TRAPPC11*, *TRPC3*, *TRPV4*, *TSEN2*, *TSEN34*, *TSEN54*, *TSFM*, *TTBK2*, *TTC19*, *TTPA*, *TUBB2B*, *TUBB4A*, *TWNK*, *UBAP1*, *UBQLN2*, *UBTF*, *USP8*, *VAMP1*, *VAPB*, *VARS2*, *VCP*, *VLDLR*, *VPS13A*, *VPS13D*, *VPS37A*, *VPS53*, *VRK1*, *VWASB*, *WASHC5*, *WDR45*, *WDR48*, *WDR73*, *WDR81*, *WFS1*, *WWOX*, *XPA*, *XPC*, *ZFR*, *ZFYVE26*, *ZFYVE27*, *ZNF423*,* ZNF592*

The recommendations in Table 2 work best in cross-sectional database explorations of large patient collections and are based on experiences with successful gene discoveries across NGS data sharing platforms (GENESIS, RD-Connect GPAP, and CAGC) containing ataxia datasets.

### Prioritization

Prioritization of filtered variants can be guided by scores for automated variant prioritization/ranking (often machine-learning [ML]-based such as Exomiser and MAVERICK). While each of these scores can, of course, also be used for variant filtering, we mostly use them in their capacity to provide an important addition to the prior rule-based evaluation of genetic variation, here having demonstrated great success at prioritizing variants in AGI NGS platforms [18, 19]. These scores are used in tools such as MAVERICK and Exomiser, which can be executed from GENESIS and RD-Connect GPAP respectively. Both tools provide a variant prioritization/ranking based on genetic (Exomiser and MAVERICK) and phenotypic information (Exomiser only) for protein-altering SNVs and small indels, which is highly valuable in a family-based analysis [18, 19]. Both Exomiser and MAVERICK are capable of consistently ranking the causative pathogenic variant within the top five variants in over 80% and 95% of cases respectively [18, 19].

## Consensus Recommendations for Standardized Clinical Metadata Collection

Capturing the clinical metadata in a standardized way, which is harmonized across platforms is a keystone of rare disease data sharing: (i) provide clinicians with standardized phenotype collection and (ii) that can be submitted into multiple platforms without additional effort. The clinical metadata of the individuals exist in parallel to the NGS data. They should provide standardized machine-readable information on:

- the disease status (affected/unaffected)
- the sex of the individual
- presumed inheritance pattern
- pedigree
- links to records of family members
- consanguinity between family members
- ethnicity
- patient’s phenotype

The ataxia data sharing platforms GENESIS and RD-Connect GPAP use ORDO (Orphanet Rare Disease Ontology) as the general disease nomenclature (e.g. ORPHA:99 code; autosomal dominant cerebellar ataxia). ORDO has been recognized as the most appropriate nomenclature for clinical coding of rare diseases in Europe by the European Commission Expert Group on Rare Diseases [20]. In addition, the three platforms rely on the Human Phenotype Ontology (HPO) to describe clinical phenotype abnormalities in separate standardized terms (e.g. HP:0,002,378 term; Hand tremor) [21]. Using common and machine-readable ontologies facilitates automated analyses and data transfers and submissions between and to multiple platforms.

The standardized coding of patient clinical information makes it possible to assess similarities and differences between patients when evaluating potentially causal genetic variations. Additionally, one of the main strengths of standardized coding in data sharing platforms is the ability to use this coding to build and analyze cohorts of phenotypically similar (ataxia) patients. This not only allows to contribute to matchmaking efforts to match phenotypically similar patients with genetically similar variants between platform users [22]. In particular, it also allows to establish cohorts sufficiently standardized for rare variant burden analysis (RVAS). Given that the numbers of promising large ataxia families still unsolved is getting smaller, such novel ways for gene hunting by aggregating small families and even simplex cases is one of the next steps in ataxia NGS gene-hunting. RVAS approaches have shown to unravel promising novel variants even in rare neurological disease cohorts as small as *n* = 343 and *n* = 515 patients, as recently demonstrated for CMT and HSP, respectively [23].

## Consensus Recommendations for Ataxia NGS Data Sharing

As a multi-site collaborative consortium, all partners of the Ataxia Global Initiative were invited to submit NGS data to any of the three associated platforms, while not restricting submission to other external NGS platforms. Agreements between the associated platforms were established to ease matchmaking efforts, through contact with specified correspondence partners, and to ease NGS data transference and discovery of duplicate data between the platforms. The three AGI-associated platforms for ataxia NGS analysis—GENESIS, RD-Connect GPAP, and CAGC—all provide preset genetic filters as well as the freedom to build and save custom filters, providing easy access and essential adaptability for clinicians and geneticists alike. In addition to family-based variant filtering, all three platforms provide ways to perform cohort-based and gene-based filtering, investigating enrichment of specific variants or phenotypes. While the platforms strive towards a shared goal, the platforms develop separately, supporting different tools, and analysis pipelines. We therefore recommend submitting data to multiple platforms in a transparent way. It is also important to note that the AGI associated platforms are particularly enriched for those patients that are challenging to solve genetically. These challenging cases will benefit from the advanced analysis tools available and the genetic matchmaking potential with non-AGI data and related phenotypes present on these platforms. A prior negative result in clinical testing does not mean causative genetic variants lie outside of the data previously produced. In fact, a pathogenic variant might evade recognition due to bioinformatics analysis approaches, incomplete phenotypic or family information, as well as lack of specific annotations of identified genes and variants. Therefore, research-based analysis and reanalysis, as provided by the AGI platforms, is essential for supplementing clinical diagnostic efforts.

*GENESIS* hosts > 17.000 rare disease datasets of which > 2.000 are ataxia datasets. Since 2011, GENESIS has contributed to the discovery of > 17 ataxia new genes. Moreover, GENESIS has shown to be an effective matchmaker for gene and variant discoveries. Some of the key GENESIS features include an intuitive functional and conservation scoring system (1–5 stars), and the development and implementation of artificial intelligence tools for Mendelian variant (MAVERICK) and STR variant (RExPRT) prioritization [19]. It also includes innovative algorithms for CNV (Breakdancer, Breakseq, CNVnator, Delly, Lumpy, and Manta) and STR analysis (ExpansionHunter, ExpansionHunter DeNovo, REViewer).

*RD-Connect GPAP* hosts nearly 28,000 rare disease datasets of which nearly 1,900 are ataxia datasets, supporting its role in the discovery of > 16 ataxia genes since 2016. The RD-Connect GPAP is a node of the MatchMaker Exchange Network, allowing it to easily discover similar datasets within other large data collections. Furthermore, integration of Exomiser makes it easier for users to prioritize variants based on HPO terms associated with variants and genes [18, 22].

*CAGC* (Centralized Ataxia Genomics Core), started in 2020, currently hosts > 850 ataxia datasets and has already contributed to the discovery of *TRPC3* as a novel ataxia gene. CAGC uniquely offers Health Insurance Portability and Accountability Act (HIPAA) compliance for centers that consider genomic sequencing protected health information (PHI). The CAGC pipeline includes innovative algorithms for CNV (CoNIFER, XHMM, HMZDelFinder) and STR analysis (STRetch, ExpansionHunter, ExpansionHunter DeNovo).

The AGI associated platforms are successful models of how data sharing within the field of inherited ataxias and the broader rare disease field are enabling improved international collaborations between clinicians, geneticists, and molecular biologists. Upholding a code of conduct for data sharing is an important part of NGS data sharing within the AGI community that all AGI participants and particularly the AGI-associated platforms are striving towards. The AGI-platforms are already adhering to most of the proposed international code of conduct standards as proposed by Matar et al. [24], regarding data collection, data storage, data sharing/transfer/access, and public and community engagement, but strive to implement measures fitting the code of conduct regarding compelled disclosures and establishing a governance system.

NGS data sharing platforms are an essential part of establishing genetic ataxia diagnoses for patients and achieving technical advances for NGS analysis, providing the field with new tools for the repurposing of NGS for STR, CNV, and SV analyses as well as the development and rigorous testing of variant prioritization tools. The increasing efforts of these platforms supports the identification of druggable (gene) targets, trial-readiness, and natural history studies. Furthermore, they provide clinicians an accessible way to analyze patient data and to connect with scientists, as well as to support international connections and matchmakings on patient, gene, and variant levels.

## Outlook

Due to the increased burden of repeat expansion disorders in the ataxia field, especially when compared to other neurodegenerative diseases, improved repeat expansion identification will be a major step forward. The broader ataxia community and the major NGS data sharing platforms, have started to address this need by implementing or developing dedicated tools for the identification of repeats in short-read NGS, but also by accepting long-read WGS data, in which repeat expansion detection will be easier and more reliable.

A fast-track approach to novel targeted and mechanistic therapies is the focus on identifying causal genetic variation, such as cryptic splice variants amenable to N-of-1 ASO approaches [25]. Complementary approaches have focused on aggregating a “treatabolome,” a collection of treatable variants, ideally in a way that automatically notifies users of these treatable variants or variants in treatable genes (https://treatabolome.cnag.crg.eu) [26, 27].

Extensive data sharing of NGS datasets will have a significant effect on narrowing the diagnostic gap and the identification of the underlying genetic cause. Enabling extensive data sharing and improving the tools available will help overcome two of the main hurdles to move the ataxia genetics field forward.

## Data Availability

The platforms discussed in this manuscript can be accessed through the following webpages: GENESIS (www.tgp-foundation.org/genesis-log-in); RD-Connect GPAP (https://platform.rd-connect.eu/); CAGC (https://uclahs.fyi/cagc).
